# TET-mediated 5-methylcytosine oxidation in tRNA promotes translation

**DOI:** 10.1074/jbc.RA120.014226

**Published:** 2020-11-23

**Authors:** Hui Shen, Robert Jordan Ontiveros, Michael C. Owens, Monica Yun Liu, Uday Ghanty, Rahul M. Kohli, Kathy Fange Liu

**Affiliations:** 1 Department of Biochemistry and Biophysics, Perelman School of Medicine, University of Pennsylvania, Philadelphia, Pennsylvania, USA; 2 Graduate Group in Biochemistry and Molecular Biophysics, Perelman School of Medicine, University of Pennsylvania, Philadelphia, Pennsylvania, USA; 3 Department of Medicine, Perelman School of Medicine, University of Pennsylvania, Philadelphia, Pennsylvania, USA

**Keywords:** 5-methylcytocine, transfer RNA, translational regulation, demethylation, RNA modification, BSA, bovine serum albumin, FBS, fetal bovine serum, LC-MS/MS, liquid chromatography and tandem mass spectrometry, mESCs, mouse embryonic stem cell, m^5^C, 5-methylcytosine, NSUN2, NOP2/Sun domain protein 2, PBST, 1× phosphate-buffered saline, 0.1% Tween, SSC, saline-sodium citrate, TET, ten-eleven translocation, TET2-CD, TET2 catalytic domain, tRNA, transfer RNA, TRDMT1, tRNA aspartic acid MTase1, 5hmc, 5-hydroxymethylcytosine, 5fc, 5-formylcytosine, 5caC, 5-carboxylcytosine

## Abstract

Oxidation of 5-methylcytosine (5mC) in DNA by the ten-eleven translocation (TET) family of enzymes is indispensable for gene regulation in mammals. More recently, evidence has emerged to support a biological function for TET-mediated m^5^C oxidation in messenger RNA. Here, we describe a previously uncharacterized role of TET-mediated m^5^C oxidation in transfer RNA (tRNA). We found that the TET-mediated oxidation product 5-hydroxylmethylcytosine (hm^5^C) is specifically enriched in tRNA inside cells and that the oxidation activity of TET2 on m^5^C in tRNAs can be readily observed *in vitro*. We further observed that hm^5^C levels in tRNA were significantly decreased in *Tet2* KO mouse embryonic stem cells (mESCs) in comparison with wild-type mESCs. Reciprocally, induced expression of the catalytic domain of TET2 led to an obvious increase in hm^5^C and a decrease in m^5^C in tRNAs relative to uninduced cells. Strikingly, we also show that TET2-mediated m^5^C oxidation in tRNA promotes translation *in vitro*. These results suggest TET2 may influence translation through impacting tRNA methylation and reveal an unexpected role for TET enzymes in regulating multiple nodes of the central dogma.

Along with the post-translational modifications of histone proteins, the direct, reversible methylation of cytosines in CG dinucleotides in DNA (called CpG sites) is one of several layers of regulatory information that determines chromatin state (1, 2, 3). The ten-eleven translocation (TET) family of 5-methylcytosine dioxygenases catalyzes the successive oxidation of 5-methylcytosine (abbreviated as “5mC” in DNA) to 5-hydroxymethylcytosine (5hmC), to 5-formylcytosine (5fC), and lastly to 5-carboxylcytosine (5caC), providing an additional layer of epigenetic regulation to the mammalian genome (4, 5, 6). Biochemical assays suggest that one member of the TET family, TET2, works on both DNA and RNA as well (7), and recent findings have begun to reveal the biological function of TET-mediated oxidation in RNA. One study suggested that TET-mediated oxidation in mRNA promotes global protein synthesis in *Drosophila* (8). Several earlier studies have also shown that TET-mediated mRNA oxidation decreases stability. These effects may result from ADAR1-mediated repression of the target genes (9). Conversely, 5-methylcytosine in RNA (abbreviated as m^5^C) can also promote mRNA stability through specific m^5^C reader proteins (10, 11). m^5^C oxidation has been postulated to disrupt the binding of these m^5^C-specific readers and thereby tune stability.

While the abundance and occupancy of m^5^C sites in mRNA remain under investigation, m^5^C is highly abundant in tRNA. The majority of known functional roles of m^5^C in RNA species are also from the studies of m^5^C sites in tRNAs. In tRNA, m^5^C sites occur most often at the junction of the variable loop and the T stem-loop. The modification of three cytosines spanning positions 47 to 5-0 has been suggested to stabilize the tRNA structure (12, 13). Cytosine 38 in the anticodon loop of the tRNA is another frequently modified site. m^5^C38 in mouse tRNA^Asp^ has been shown to stimulate amino acid charging of the tRNA and to facilitate translation of poly-Asp–containing proteins (14). In addition, m^5^C38 can protect tRNAs from stress-induced endonuclease-mediated fragmentation (15, 16) and help to maintain correct translational read-out of near-cognate codons (17). m^5^C also exists at C34 in tRNA^Leu(CAA)^ and mitochondrial (mt) tRNA^Met^ (18, 19) and mt-tRNA^Met^ in mammals (20, 21, 22). Lastly, m^5^C installation has been shown to be important for the final steps of tRNA^Thr^ and tRNA^Cys^ biogenesis (23). In addition to its well-documented functions in tRNA, m^5^C also exists in rRNA and is important for translational fidelity (24, 25, 26). The m^5^C sites in RNA are installed by several methyltransferases, including tRNA aspartic acid MTase1 (TRDMT1), Dnmt2, NOP2/Sun domain protein 2 (NSUN2), Nsun3 (20, 21), and Nsun6 (27). The biological significance of m^5^C in RNA is further emphasized by genetic studies. For instance, knockout of NSUN2 in mice causes male infertility and reduced growth (28), while mutations in human NSUN2 are involved in intellectual disability (29). Lastly, DNMT2 deficiency has also been shown to affect polypeptide synthesis in humans (16).

Interestingly, cytoplasmic and mitochondrial tRNAs have been shown to carry f^5^C, an oxidation product of m^5^C. The alpha-ketoglutaric acid–dependent dioxygenase ALKBH1 has been shown to be involved in the biogenesis of f^5^C at the first position of the anticodon (position 34 of canonical tRNAs) in mitochondrial tRNA^Met^ (20, 30). f^5^C of mt-tRNA^Met^ is important for the decoding of AUA methionine codons during mitochondrial translation (31). Additionally, ALKBH1 had also been shown to catalyze the formation of 5-hydroxymethyl-2-O-methylcytidine (hm^5^Cm) and 5-formyl-2-*O*-methylcytidine (f^5^Cm) at the same position in cytoplasmic tRNA^Leu^ (32). ALKBH1-catalyzed oxidation reactions are important for translation and mitochondrial function. However, m^5^C oxidation in tRNA^Leu^ and mitochondrial tRNA^iMet^ is specifically carried out by ALKBH1, not the TET enzymes (30). Whether TET-mediated m^5^C oxidation occurs on tRNA species as well as their biological functions are not fully understood. All these previous studies have highlighted the importance of the reversible regulation of m^5^C in tRNA. Here, we investigated whether TET2 can catalyze tRNA m^5^C oxidation and the potential biological function from this oxidation reaction.

## Results

**Figure 1:**
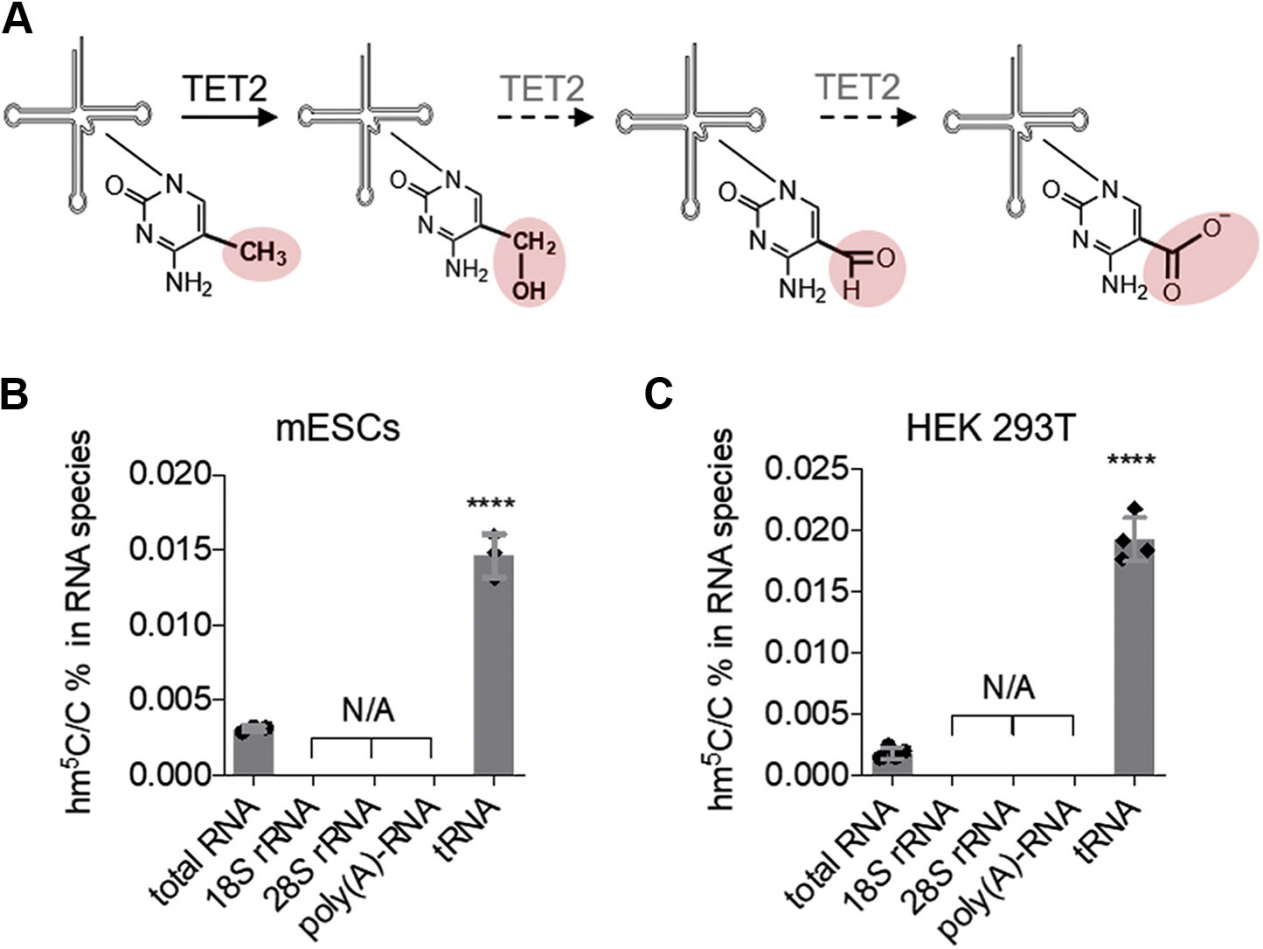
**hm**^**5**^**C is enriched in tRNA**. *A*, schematic of TET2-mediated m^5^C oxidation products. *B*, LC-MS/MS quantification of hm^5^C in total RNA, 18S rRNA, 28S rRNA, poly(A)-RNA, and tRNA extracted from mESCs. *C*, LC-MS/MS quantification of hm^5^C in total RNA, 18S rRNA, 28S rRNA, poly(A)-RNA, and tRNA extracted from mESCs HEK293T cells. *p* values were determined using two-tailed Student's *t* test for unpaired samples. Error bars represent mean ± s.d., n = 4 (four biological replicates × two technical replicates) ∗∗∗∗ *p* < 0.001. LC-MS/MS, liquid chromatography and tandem mass spectrometry; mESCs, mouse embryonic stem cells.

To study whether TET enzymes catalyze oxidation on m^5^C in other RNA species, we first quantified the levels of hm^5^C (the first m^5^C oxidation product, Fig. 1*A*) in several major RNA species. We extracted total RNA, 18S rRNA, 28S rRNA, polyadenylated RNA (poly(A)-RNA), and tRNA from mouse embryonic stem cells (mESCs) and HEK 293T cells (Fig. S1, *A*–*D*). The extracted RNAs were then degraded to single nucleosides before being analyzed via triple quadrupole liquid chromatography and tandem mass spectrometry (LC-MS/MS). The LC-MS/MS results show that among these different RNA species, hm^5^C is more significantly enriched in tRNAs in both mESCs and HEK293T cells (Fig. 1, *B*–*C*, Fig. S1*E*, and Fig. S2).

**Figure 2:**
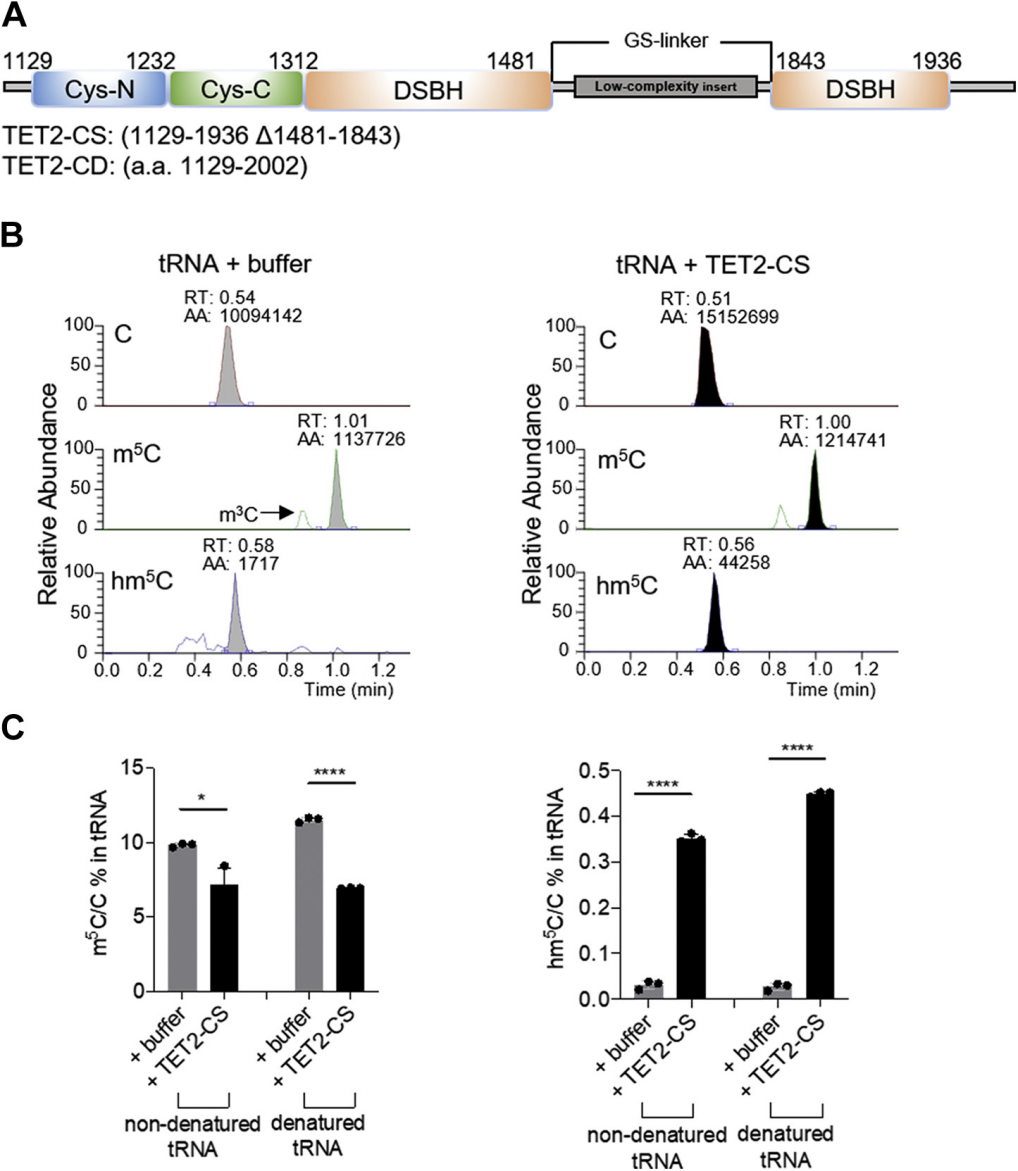
**TET2-CS–mediated *in vitro* oxidation reaction**. *A*, constructs of TET2-CS and TET2-CD. *B*, LC-MS/MS tracks of C, m^5^C, and hm^5^C from *in vitro* oxidation reaction of TET2-CS with purified tRNAs. tRNAs purified from HEK293T cells were incubated with either TET2-CS or buffer in the presence of alpha-ketoglutarate. *C*, LC-MS/MS quantification of C, m^5^C, and hm^5^C from *in vitro* oxidation reaction of TET2-CS with purified tRNAs. *p* values were determined using two-tailed Student's *t* test for unpaired samples. Error bars represent mean ± s.d., n = 3 (three biological replicates × two technical replicates). ∗ *p* < 0.05, ∗∗∗∗ *p* < 0.001. AA, atomic absorption; Cys-C, cysteine (Cys)-rich domain C terminal; Cys-N, cysteine (Cys)-rich domain N terminal; DSBH, double-stranded β-helix; LC-MS/MS, liquid chromatography and tandem mass spectrometry; N/A, not detectable; RT, retention time.

In light of recent discoveries that TET2 may be responsible for modifying RNA in cells (33), we focused on the role of TET2 in accounting for the observed enrichment of hm^5^C in tRNA. To investigate if m^5^C can be oxidized by TET2, we conducted *in vitro* oxidation assays. The activity of a purified TET2 truncation variant (TET2-CS, previously crystallized (34), Fig. 2*A*) was first confirmed on a 5mC-containing ssDNA oligo (Fig. S3, *A*–*C*). We then incubated tRNAs purified from HEK293T cells either with TET2-CS or in buffer alone. To investigate whether tRNA structure has an impact in TET-mediated oxidation, we used both native and denatured tRNAs in this biochemical oxidation reaction. Analysis of the oxidation products via LC-MS/MS clearly shows that TET2-CS is capable of oxidizing m^5^C to hm^5^C in tRNA *in vitro* (Fig. 2, *B*–*C*), while the further oxidation products, f^5^C and ca^5^C, were not detected (Fig. S3, *D*–*E*). However, in the presence of EDTA (an iron chelator), TET2-CS cannot lead to decreased m^5^C and increased hm^5^C in tRNA (Fig. S4).

**Figure 3:**
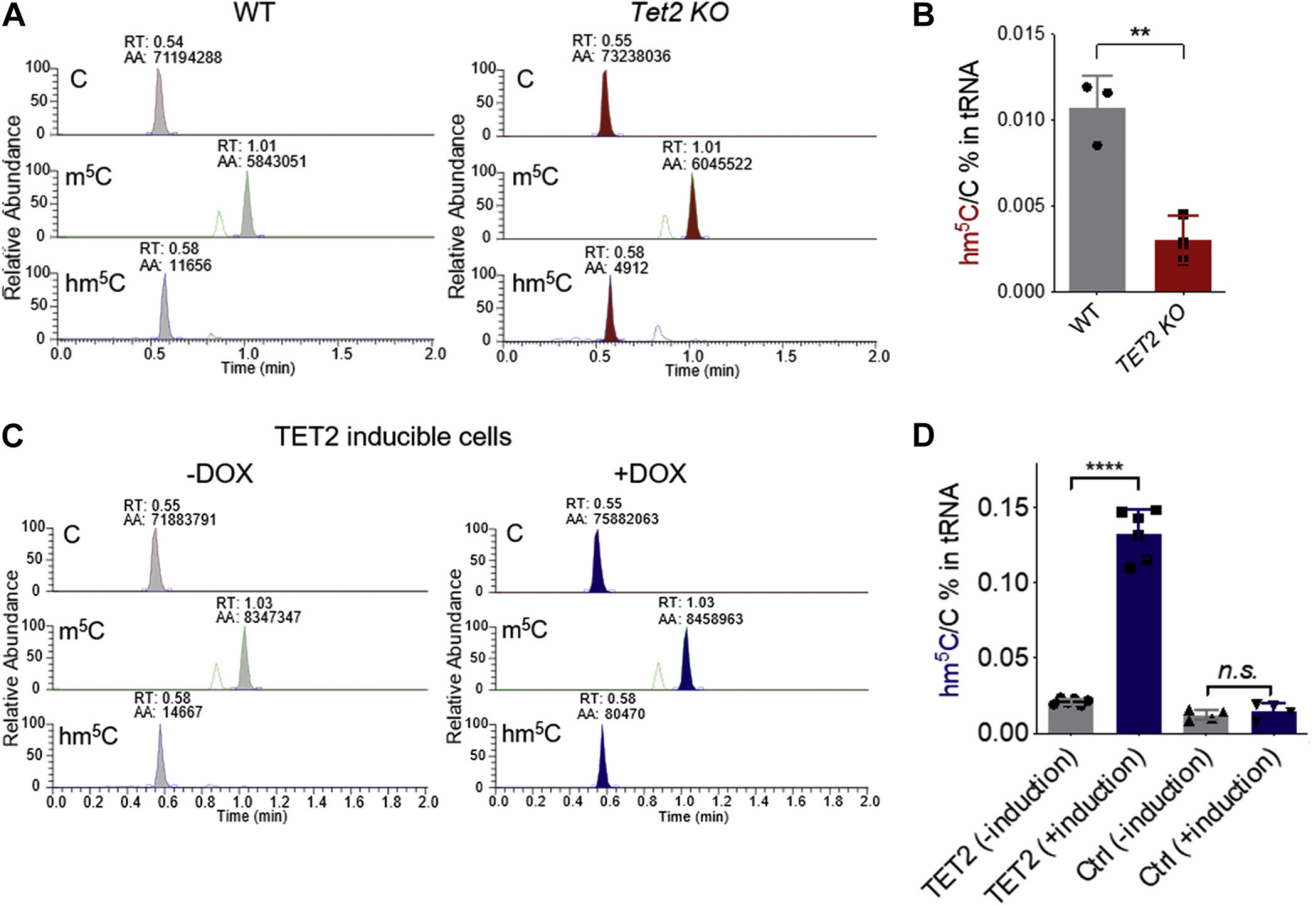
**TET2-mediated m**^**5**^**C oxidation inside cells**. *A*, LC-MS/MS tracks of C, m^5^C, and hm^5^C from purified tRNAs extracted from wild-type and *Tet2* KO mESCs. *B*, LC-MS/MS quantification of hm^5^C in tRNA extracted from wild-type and *Tet2* KO mESCs. *C*, LC-MS/MS tracks of C, m^5^C, and hm^5^C from purified tRNAs extracted from TET2-inducible expression HEK293T cells before and after doxycycline induction. *D*, LC-MS/MS quantification of hm^5^C in tRNA extracted from TET2 inducible expression HEK293T cells and control HEK293T cells before and after doxycycline induction. *p* values were determined using two-tailed Student's *t* test for unpaired samples. Error bars represent mean ± s.d., n = 3 (three biological replicates × two technical replicates) ∗∗ *p* < 0.01, ∗∗∗∗ *p* < 0.001, n.s. means *p* > 0.05. AA, atomic absorption; KO, knock-out; LC-MS/MS, liquid chromatography and tandem mass spectrometry; mESCs, mouse embryonic stem cells; RT, retention time.

Given that we observed that the enrichment of hm^5^C in tRNA and that TET2 is capable of generating this modification *in vitro*, we sought to study if TET2-mediated oxidation generates tRNA hm^5^C in cells. To this end, we quantified m^5^C and hm^5^C levels in tRNAs extracted from wild-type and *Tet2* KO mESCs (Fig. 3). The results show that *Tet2* KO leads to a significant decrease of hm^5^C in tRNA. We also observed a noticeable, but not significant, increase of m^5^C in tRNA in *Tet2* KO mESCs in comparison with the wild-type mESCs (Fig. 3, *A*–*B* and Fig. S5*A*). Although the expression of TET2 in mESCs leads to the starkly obvious increases of hm^5^C in tRNAs, we observed a low level of hm^5^C in tRNA. These results suggest that TET2 does not demethylate all m^5^C sites in tRNAs. It is possible that TET2 works with other demethylase enzymes such as ALKBH1 (30) to work on individual subsets of tRNAs.

To further confirm that TET2 is responsible for tRNA m^5^C oxidation in cells, we first constructed a doxycycline-inducible expression system for the TET2 catalytic domain (TET2-CD) in HEK293T cell line (35). Using this expression system, TET2-CD expression can be titrated to near-physiological expression levels, thereby avoiding any potential artifacts caused by overexpression (Fig. 2*A* and Fig. S5, *B*–*C*). We then validated that this increase in TET2-CD expression resulted in an increase in TET2-CD activity. To do this, we performed dot-blot assays using anti-5mC and anti-5hmC antibodies to further validate our LC-MS/MS findings (Fig. S5*D*). In addition, we quantified and compared 5mC and 5hmC levels in DNA in our cells before and after doxycycline treatment using LC-MS/MS. The results show that induced expression of TET2-CD leads to decreased levels of 5mC and increased 5hmC levels in DNA (Fig. S5, *E*–*F*). These results were consistent with the results from the dot-blot assay and together collectively suggested that our doxycycline-inducible system can successfully induce the expression of functional TET2-CD. After we validated the inducible cell line, we next quantified m^5^C and hm^5^C levels in tRNA before and after doxycycline treatment. The LC-MS/MS results show that induced expression of the catalytic domain of TET2 leads to significantly increased hm^5^C levels in tRNA (Fig. 3, *C*–*D*) with no obvious change of m^5^C in tRNA (Fig. S5*G*). We reasoned that it is due to the higher level of m^5^C in comparison with hm^5^C in tRNA; also TET2 is not the only tRNA m^5^C demethylase. In contrast, the parental HEK293T cell line (which we used to construct the inducible cell line) did not show any change of both m^5^C and hm^5^C levels before and after doxycycline treatment (Fig. 3*D* and Fig. S5, *G*–*H*). Together with the results from *Tet2* KO mESCs, these results revealed that TET2-mediated m^5^C oxidation occurs on tRNA inside cells.

**Figure 4:**
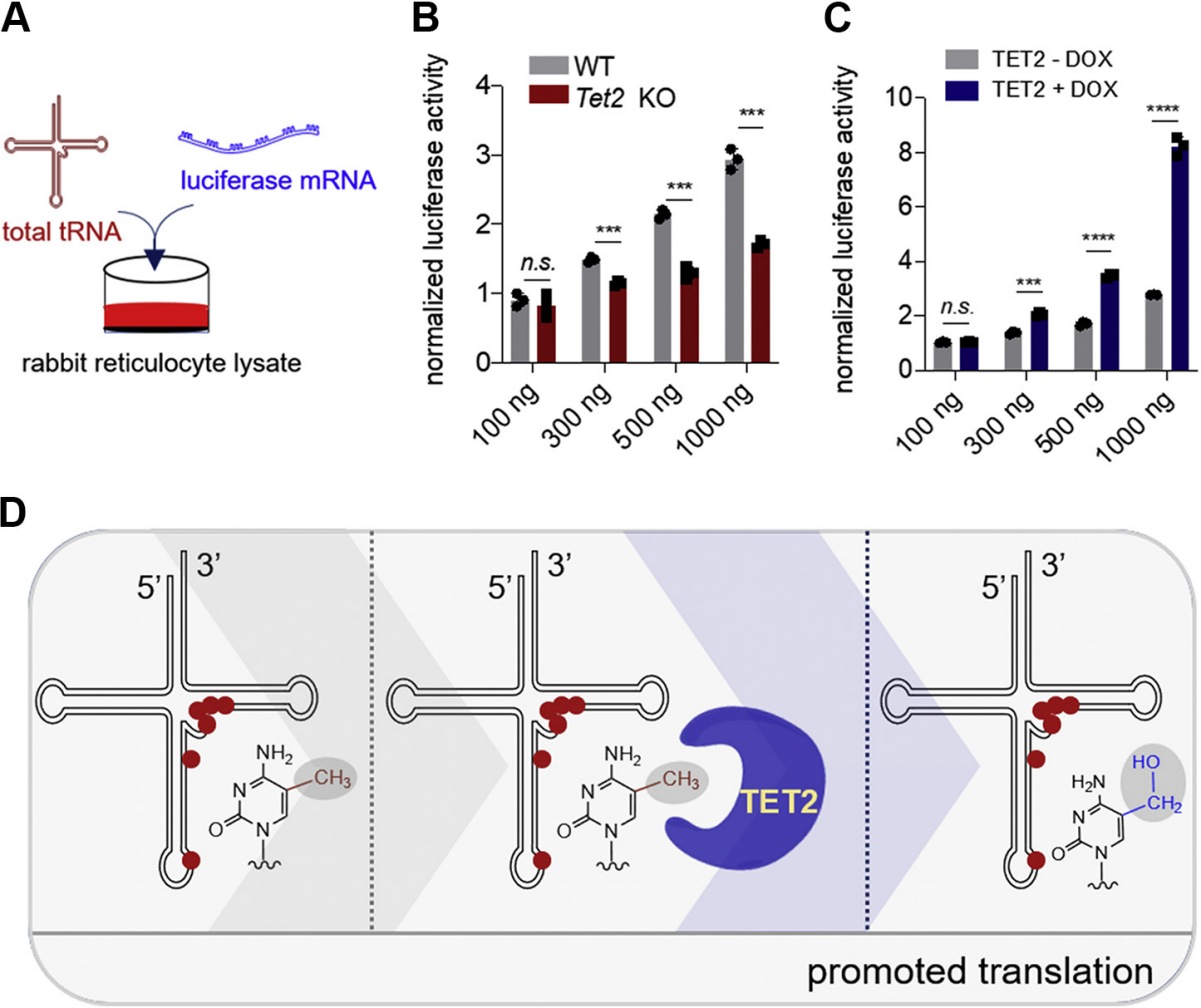
**TET2-mediated tRNA oxidation significantly promotes translation**. *A*, illustration of the *in vitro* translation assay. *B*, quantification of luciferase signals from the *in vitro* translation system supplemented with 100 to 1000 ng of tRNAs extracted from wild-type and *Tet2* KO mESCs. *C*, quantification of luciferase signals from the *in vitro* translation system supplemented with 100 to 1000 ng of tRNAs extracted from HEK293T cells with inducible TET2-CD before and after doxycycline induction. Error bars represent mean ± s.d., n = 3 (three biological replicates × two technical replicates). *D*, illustration of TET2-mediated m^5^C oxidation promotes translation. The previously confirmed m^5^C sites (C34, C38, C47-C50) in tRNA are highlighted as *red balls*. KO, knock-out; mESCs, mouse embryonic stem cells; TET2-CD, TET2 catalytic domain.

After observing tRNA as a target for TET-mediated oxidation, we investigated the possible biological consequences of TET2-mediated oxidation on m^5^C in tRNA. Given that tRNA modifications can tune translational efficiency (36), it is possible that TET-mediated oxidation of tRNAs could affect translation. To investigate this hypothesis, we utilized a rabbit reticulocyte-based *in vitro* translation system to measure the production of active luciferase protein from a fixed amount of luciferase mRNA (Fig. 4*A*). To probe how TET2-mediated oxidation of tRNAs affects translation, we first extracted tRNAs from either wild-type or *Tet2* KO mESCs. We then spiked-in increasing amounts (100, 300, 500, and 1000 ng) of purified tRNAs into separate translation reactions along with the luciferase mRNA (uncapped *in vitro*-transcribed RNA containing a 30-base poly(A) tail from Promega) and measured luciferase activity. The results show that tRNAs originating from wild-type mESC cells lead to significant increase of luciferase activity. In contrast, spiking in tRNAs extracted from the *Tet2* KO cells did not lead to increased luciferase signals (Fig. 4*B*). We also performed the *in vitro* translation assays using tRNAs extracted from TET2-inducible expression cells before and after administration of doxycycline. The results show that tRNAs originating from cells after doxycycline addition lead to an obvious increase of luciferase signal whereas the tRNAs from untreated cells elicit a relatively lower luciferase signal (Fig. 4*C*). In this *in vitro* translation reaction, we can rule out the possibility that TET2 proteins bind to the luciferase mRNA to promote translation since the reaction system only contains supplemented tRNAs, luciferase mRNA, and reticulocytes. Furthermore, we studied whether the expression of TET2 leads to increased translation inside cells. To this end, we quantified protein synthesis in TET2-induced and uninduced conditions using puromycin incorporation followed by Western blot analysis. The results showed that the expression of TET2 did not lead to an obvious impact on overall translation (Fig. S6*A*). It would be insightful to investigate the impact of TET2-mediated tRNA oxidation on specific transcripts in future studies.

We also sought to identify the specific tRNA targets of TET2. Since NSUN2 is one of the most studied tRNA m^5^C methyltransferase enzymes, we examined whether TET2 works on the m^5^C-containing tRNA targets of NSUN2 (16). As shown in updated Figure S6*B*, we quantified the levels of m^5^C and hm^5^C levels in four tRNAs extracted from *Tet2* KO and wild-type mESCs. The results showed that *Tet2* KO leads to a significant increase of hm^5^C and a noticed decrease of m^5^C level in tRNA^Gly^ (Fig. S6*B*). The effects of TET2-mediated oxidation are not obvious on the other three tRNAs including tRNA^Asp^, tRNA^Val^, and tRNA^Leu^ (Fig. S6*B*). These results collectively suggested that tRNA^Gly^ is a possible target of TET2.

## Discussion

The presence of an appreciable level of hm^5^C in cellular RNA and the involvement of the TET family of enzymes in producing this modification support the hypothesis that the function of TET enzymes is not restricted to epigenetic regulation at the DNA level. Our results suggest that TET2 can oxidize m^5^C and generate hm^5^C on tRNAs in mammalian cells. We show that hm^5^C is particularly abundant in tRNAs in comparison with other major RNA species including rRNA and poly(A)-RNAs. This suggests that tRNA is possibly a major RNA target of TET2.

Our finding that TET2 can oxidize m^5^C on tRNA inside cells raises questions regarding the localization and timing of this activity. Given that TET2 is known to localize to the nucleus (37), it is likely that the oxidation of tRNAs is performed in that compartment as a step in pre-tRNA maturation following transcription and m^5^C installation by NSUN2/DNMT2 (16). These nascent pre-tRNAs would likely present accessible m^5^C sites, some of which may become occluded once fully folded and mature. While pre-tRNAs are the most likely primary tRNA substrate of the TET2-CD in our system, this is not the only possibility. Mature tRNAs are known to be transported into the nucleus via a retrograde importation mechanism (38, 39, 40); thus, it is also possible that mature tRNAs are a substrate of the TET2-CD.

As our results suggest, the decrease of m^5^C levels in tRNA upon expression of the TET2-CD is reproducible yet not significant while the increase of hm^5^C in tRNA is significant, (Fig. 3, *C*–*D* and Fig. S7*G*). This implies that only a subset of tRNAs and possibly only specific but not all m^5^C sites in tRNAs are the targets of the TET2-CD in our system, a hypothesis which is supported by the previous findings of ALKBH1-mediated tRNA m^5^C oxidation (30). Furthermore, whether TET1 and TET3 can oxidize m^5^C in tRNAs is unknown. Given that TET3 resides in both the cell nucleus and the cytoplasm (41), cytoplasmic TET3 might have optimal accessibility to mature tRNAs. It is possible that tRNA m^5^C sites are under special and temporal regulation. Along the tRNA maturation process, it is possible that several demethylase enzymes can work on different tRNA m^5^C sites in both the nucleus and the cytoplasm to facilitate proper tRNA biogenesis and regulation. In addition, it is known that reprogramming of m^5^C34 in tRNA facilitates translation of specific ribosomal proteins upon oxidative stress to sustain life (42). The dynamic regulation of the individual sites of m^5^C in tRNAs by distinct enzymes may represent another layer of translational control, especially when faced with different types of environmental stresses. This work provides a starting point for future studies to determine which tRNAs are targeted by TET2, what effects these oxidized tRNAs exert on translation, and how target and nontarget tRNAs are discriminated by TET2.

Lastly, while we were able to see a clear enrichment of hm^5^C in tRNAs, further oxidation products (namely f^5^C and ca^5^C) were not detectable in our analyses. Given that the role of these modifications in DNA is currently a matter of research (43), their apparent absence in tRNA in our system raises questions about TET2's activity on RNA. We have previously described two important characteristics of TET activity (7): (1, 7) DNA is generally the preferred substrate as opposed to RNA for TET oxidation, and (2) the first oxidation step (m^5^C to hm^5^C) is the most efficient and preferred step, while f^5^C and ca^5^C are less efficient products in both DNA and RNA. These two points may explain why the further oxidation products are absent in tRNA; the disfavored substrate type compounded with the disfavored products result in an extremely low efficiency of f^5^C and ca^5^C. Moreover, the lack of further oxidative products could be due to a number of additional biological factors, ranging from the more structured nature of tRNA than that of DNA to possible steric hindrance from other tRNA-modifying enzymes that install the plethora of modifications present on mature tRNA, or possibly from other tRNA modifications themselves.

Taken together, this study reveals that tRNAs are a target for TET-mediated oxidation of m^5^C to hm^5^C and that this conversion may play a role in supporting mRNA translation (Fig. 4*D*). These findings provide a starting point for future investigations into the effects of TET2-mediated oxidation of m^5^C in tRNAs, the mechanism by which this oxidation modulates translation, and, more generally, expanding our view of the regulatory controls exerted by TET family enzymes across both epigenetics and epitranscriptomics.

## Experimental procedures

### Mammalian cell culture and plasmid transfection

HEK293T cells were cultured with Dulbecco's Modified Eagle Medium (GIBCO) media supplemented with 10% fetal bovine serum (FBS) (GIBCO), 1% Pen/Strep (GIBCO). mESCs were cultured with Dulbecco's Modified Eagle Medium (GIBCO) media supplemented with 10% FBS (GIBCO), 1% pen/strep (GIBCO), 1× non-essential amino acid (GIBCO), 1× L-glutamine (GIBCO), 50 μM 2-mercaptoethanol (Bio-Rad), and 1000 μ/ml mouse leukemia inhibitory factor (LIF) (Gemini). All the cells were cultured in a humidified cell culture incubator with 5% CO_2_ at 37 °C. For mESCs culture, MEF feeder cells (Sigma) were used. For passaging, cells were washed with PBS once, and then 0.25% trypsin was added and incubated at 37 °C for 3 min. After medium was added to inactivate trypsin digestion, cells were split for different purposes. *Tet2* KO mESC cell line was derived as previously described (44).

### Doxycycline-induced TET2-CD expression cell line construction

To generate stable cell lines capable of doxycycline-inducible TET2 expression, we used high-efficiency, low-background (HILO) recombination-mediated cassette exchange (RMCE) technology (35). This started with a plasmid containing a RIPE cassette that consists of a tetracycline-inducible EGFP gene with a puromycin resistance marker. The human TET2 catalytic domain (residues 1129–2002) was cloned in place of EGFP to yield a TET2-RIPE construct. These constructs could be transfected into HILO acceptor cells, which are variants of HEK293T cells that contain a matching recombination locus encoding blasticidin resistance. The acceptor cells were maintained in media containing DMEM, high-glucose+GlutaMAX (Thermo), 10% FBS (Sigma), 1 mM sodium pyruvate (Thermo), 1× pen/strep (Thermo), and 2.5 μg/ml of blasticidin S (Thermo). For transfection, cells were plated into antibiotic-free medium to ∼70% confluency. Cells were then cotransfected with 990 ng of TET2-RIPE plasmid plus 10 ng of Cre recombinase plasmid, with the aim to insert the TET2-RIPE cassette into the acceptor locus via Cre-mediated recombination, thus swapping blasticidin for puromycin resistance. The next day, cells were split 1:2 and, after about 6 h, puromycin was added to a concentration of 2 μg/ml. Every 24 h, medium was changed, and the puromycin concentration was increased to 4 μg/ml for more stringent selection. Once colonies formed and began to expand, puromycin was reduced back to 2 μg/ml for maintenance.

PCR was used to confirm locus-specific recombination in the TET2 stable cell lines. Cells were harvested, and genomic DNA was extracted. Primers that flanked the region where recombination takes place were paired with primers complementary to sequences in the acceptor or TET2 cell line, but not both. GAPDH was used as a control.

#### PCR primers

EF: 5ʹ-CCAGCTTGGCACTTGATGT-3ʹ; BR: 5ʹ-TAGCCC TCCCACACATAACC-3ʹ; PR: 5ʹ-TCGTAGAAGGGGAGGT TGC-3ʹ; 4F: 5ʹ-CCAAAAGAGAGCTGCACGCTAC-3ʹ; BF: 5ʹ-GCAACGGCTACAATCAACAG-3ʹ; WR: 5ʹ-GGGCCACAAC TCCTCATAAA-3ʹ; hGAPDH_F: 5ʹ-CCTGACCTGCCGTCT AGAAA-3ʹ; hGAPDH_R: 5ʹ-CCCTGTTGCTGTAGCCAAAT-3ʹ.

For induction with doxycycline, cells were split 1 day prior into 6-well plates so that they would be 60 to 70% confluent on the day of induction. Doxycycline was dissolved in PBS and then added in equal volumes to the wells to achieve a final concentration of 100 ng/ml. Cells were harvested by trypsinization at 24, 48, or 72 h, and genomic DNA was extracted for LC-MS/MS analysis or dot blotting for 5mC and 5hmC modifications. Genomic DNA Purification Kit (Invitrogen) was used to extract the genome DNA following the manufacturer's instruction. RNA isolation was carried out as described below for cells induced 48 h.

### RNA isolation

#### Total RNA extraction

TRIzol (Invitrogen) was used to isolate total RNA following the manufacturer's instructions.

#### Poly-A RNA extraction

mRNA was extracted from the total RNA by using Dynabeads mRNA Purification Kit (Ambion) following the manufacturer's instructions. The rRNA was further removed by RiboMinus Eukaryote Kit (Invitrogen). The mRNA purity and integrity were tested by Bioanalyzer with RNA nano Chips (Agilent Technologies).

#### Small-RNA extraction

Small RNAs were extracted from total RNA using an RNA Clean and Concentrator -25 Kit (Zymo Research) following the manufacturer's protocol for small RNAs.

#### tRNA extraction

Purified tRNAs were obtained by first migrating 1 to 2 μg of small RNAs through a 6% urea-TBE polyacrylamide denaturing gel (Invitrogen). Nucleic acids were stained using SYBR Gold nucleic acid gel stain (Invitrogen). Migrated tRNAs were recovered from the gel using the ZR small-RNA PAGE Recovery Kit (Zymo Research).

#### tRNA isolation

The procedure was adapted from a previous report (45). Briefly, RNA species smaller than 200 nt were extracted from the total RNA using RNA Clean & Concentrator (RCC) Kits (Zymo Research). The tRNA fraction was further extracted from the small RNAs by using 15% TBE-Urea gel. For specific tRNA isolation, streptavidin-conjugated M-280 magnetic Dynabeads (Invitrogen) were used. Twenty microliters of beads were washed once with buffer A (10 mM Tris, pH 7.5, 2 M NaCl, 2 mM EDTA) and then resuspended in 20 μl of buffer A. Biotinylated DNA probes (200 μM), complementing with the sequences of specific tRNA, in 10 μl of water were mixed with the same volume of beads in buffer A. After incubation at room temperature for 30 min with gentle mixing, the probe-coated beads were washed four times with buffer B (5 mM Tris, pH 7.5, 1 M NaCl, 1 mM EDTA) and resuspended with 6 × saline-sodium citrate (SSC) solution. After mixing the total tRNA and probe-coated beads in 6 × SSC solution, they were incubated at 75 °C for 10 min. Then, the mixture was rotated at room temperature for 3 h to allow the annealing. The probe-coated beads were washed three times with 3 × SSC solution and twice with 1 × SSC. Finally, the tRNA was eluted three times with RNase-free water.

### Liquid chromatography and tandem mass spectrometry

The RNA and DNA samples were digested using Nucleoside Digestion Mix (NEB) at 37 °C for 2 h to get single nucleosides. Ten microliters of the sample or standard was injected into an HPLC-QQQ-MS/MS system. C18 column in reverse-phase ultra-performance liquid chromatography system was used to separate nucleosides with online mass spectrometry detection by the Altis (Thermo Fisher Scientific) QQQ triple-quadruple LC mass spectrometer in positive electrospray ionization mode with Buffer A (0.1% formic acid solution) and buffer B (30% acetonitrile in 0.1% formic acid solution). The nucleosides were quantified using retention time and the nucleoside-to-base ion mass transitions of 272.1 → 156.0 (5caC), 256.1 → 140.0 (5fC), 258.1 → 124.1 (5hmC), 242.1 → 126.1 (5mC), 228.1 → 112.1 (dC), 288.1 → 156.0 (ca^5^C), 272.1 → 140.0 (f^5^C), 274.1 → 124.1 (hm^5^C), 258.1 → 126.1 (m^5^C) and 244.1 → 112.1 (rC). All quantifications were performed by converting the peak area from the LC-MS/MS to moles using the standard curve obtained from pure nucleoside standards. Then, the percentages of m^5^C and hm^5^C to C were calculated and compared across different samples. All modifications are quantified using external calibration curves rather than labeled internal standards.

### Dot-blot assay

DNA samples were applied to an Amersham Hybond-N+ membrane, optimized for nucleic acid binding (GE Healthcare), in serial dilutions. After UV cross-linking three times in a Stratagene Stratalinker 2400 UV Crosslinker, the membrane was stained with 0.04% methylene blue in 0.5 M sodium acetate. Then, the membrane was washed with 1× phosphate-buffered saline, 0.1% Tween (PBST), blocked with 5% of bovine serum albumin (BSA) in PBST, and then incubated with 5% of BSA in 1 × PBST containing specific antibody (1:500) overnight at 4 °C. After washing three times with 1 × PBST, horseradish peroxidase (HRP)–conjugated secondary antibody (1:20,000) in 5% of BSA was used. The membrane was visualized by ECL Western Blotting Detection Kit (Thermo Fisher Scientific).

### TET2-CS expression and purification

N-terminally FLAG-tagged TET2-CS, the crystal structure variant of the enzyme (1129–1936 Δ1481–1843), was purified from insect cells as previously described (46). Briefly, the construct, with an N-terminal FLAG tag, was subcloned into a pFastBac1 vector. After generation of baculovirus, Sf9 cells were infected and expression was performed for 24 h. Cells from a 1 L culture were collected and resuspended in lysis buffer [50 mM HEPES (pH 7.5), 300 mM NaCl, and 0.2% (v/v) NP-40] containing complete, EDTA-free Protease Inhibitor Cocktail (Roche, 1 tablet/10 ml). Cells were lysed by three passes through a microfluidizer at 15,000 psi. The lysate was cleared by centrifugation at 20,000*g* for 30 min. The supernatant was then passed two or three times over a 500-μl or 1-ml packed column of anti-FLAG M2 affinity resin (Sigma), prepared according to the manufacturer's instructions. The column was washed three times with 10 ml of wash buffer [50 mM HEPES (pH 7.5), 150 mM NaCl, and 15% (v/v) glycerol]. One column volume of elution buffer [wash buffer containing 100 μg/ml 3× FLAG peptide (Sigma)] was used to elute the bound protein by incubating on the column for 10 min, and serial elution was collected until no more protein was detected by the Bio-Rad Protein Assay. The three most concentrated fractions were pooled, aliquoted, and stored at −80 °C.

### *In vitro* activity assays

The DNA or tRNA substrate was diluted to 100 nM or 200 nM, respectively, in reaction buffer [50 mM HEPES (pH 6.5), 100 mM NaCl, 1 mM alpha-ketoglutaric acid, 1 mM dithiothreitol, and 2 mM sodium ascorbate]. Denaturation of tRNAs was accomplished by heating the tRNA diluted in the appropriate volume of water at 80 °C for 5 min before immediately adding ice-cold reaction buffer. Fresh ammonium iron (II) sulfate was added to each reaction to a final concentration of 75 μM, and the purified enzyme was added lastly to the reactions at a final volume of 25 μl. The reactions were incubated at 37 °C for 1 h. DNA reactions were quenched by the addition of a premixed quenching solution [25 μl of H_2_O, 25 μl Oligo Binding Buffer (Zymo), and 100 μl of ethanol]. DNA oligonucleotide products were purified using the Zymo Oligo Clean & Concentrator kit and eluted in 10 μl of Millipore water. tRNA reactions were quenched by the addition of a premixed quenching and small-RNA extraction solution [25 μl RNA binding buffer (Zymo), 100 μl of ethanol]. Small-RNA (<200 nt) products were purified using the Zymo RNA Clean & Concentrator kit modified for small RNAs and eluted in 10 μl of Millipore water. DNA and RNA products were analyzed by LC-MS/MS.

### *In vitro* luciferase translation assays

*In vitro* translation assays were accomplished using the Flexi Rabbit Reticulocyte Lysate System (Promega L4960). Translation reactions were supplemented with 20 ng/ul of luciferase mRNA (Promega L4561) and with varying amounts of purified small RNAs or tRNAs. Using this *in vitro* translation system with purified tRNAs, we can avoid the effects from other RNA species such as rRNA. Assembled reactions were incubated at 30 °C. Reactions were tested for functional luciferase production using the standard luciferase assay kit with a luminometer (Promega L4960).

### Protein synthesis assay

The rate of global protein synthesis was determined using puromycin to label nascent peptides as described previously (47). Briefly, cells were labeled by adding 1 μM puromycin to the medium and incubating 1 h. The cells were collected and washed twice with PBS. Then, the cells were lysed by radioimmunoprecipitation assay buffer (25 mM Tris-HCl pH 7.4, 150 mM NaCl, 1% NP-40, 1 mM EDTA, 5% glycerol, and freshly added protease inhibitor). Samples were then analyzed by SDS-PAGE followed by Western blot using an anti-puromycin antibody (Sigma-Aldrich, MABE343), and GAPDH was used as loading control.

## Data availability

All other data are available from the corresponding author upon reasonable request. This session contains all data availability information in this study.

## Conflict of interest

The authors declare that they have no conflicts of interest with the contents of this article.
