# Supplementary material for: TET-mediated 5-methylcytosine oxidation in tRNA promotes translation
Source: J Biol Chem. 2020 Nov 23;296:100087. doi: 10.1074/jbc.RA120.014226 (PMC7949041; doi:10.1074/jbc.RA120.014226)
Supplement: Supplementary file 1 — Figures S1 to S6 [file mmc1.pdf]

TET-mediated 5-methylcytosine oxidation in tRNA promotes translation

Hui Shen[1†], Robert Jordan Ontiveros[1,3†], Michael C. Owens[1,3], Monica Yun Liu[1,2], Uday Ghanty[1,2], Rahul M. Kohli[1,2], Kathy Fange Liu[1*]

[1]Department of Biochemistry and Biophysics, Perelman School of Medicine, University of Pennsylvania, Philadelphia, Pennsylvania 19104, USA
[2]Department of Medicine, Perelman School of Medicine, University of Pennsylvania, Philadelphia, PA 19104, USA
[3]Graduate Group in Biochemistry and Molecular Biophysics, Perelman School of Medicine, University of Pennsylvania, Philadelphia, PA 19104, USA

[†]These authors contributed equally to this work.

*Correspondence to: liufg@penmeddcine.upenn.edu

Running title: TET2 functions as a tRNA m^5C demethylase

Keywords: 5-methylcytocine, transfer RNA, translational regulation, demethylation, RNA modification

Supporting Information

Included Supporting Information: Supplementary Figures S1-S6

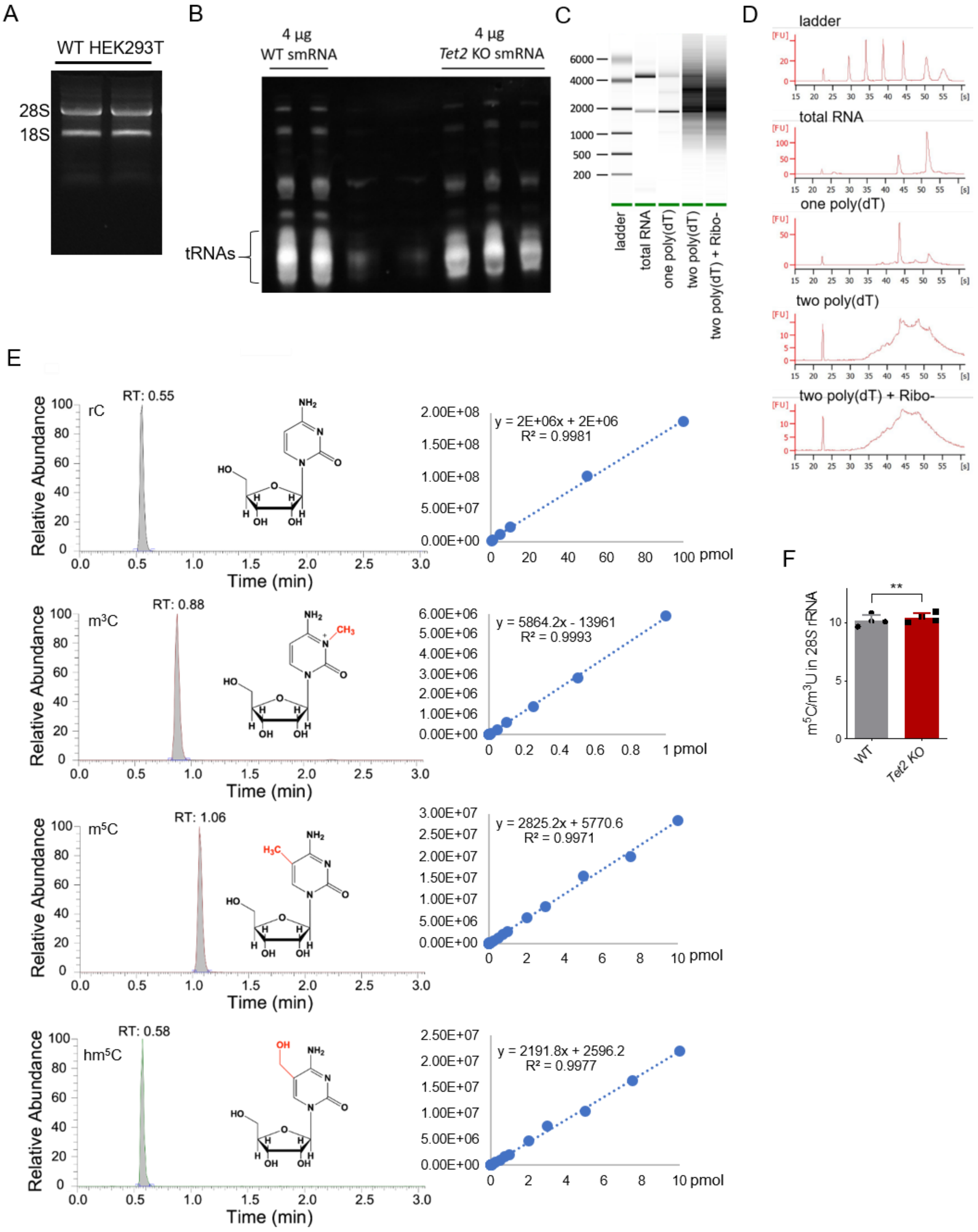
A
WT HEK293T
28S
18S
B
4 µg WT smRNA
4 µg Tet2 KO smRNA
tRNAs
C
6000
4000
2000
1000
500
200
ladder
total RNA
one poly(dT)
two poly(dT)
two poly(dT) + Ribo-
D
ladder
total RNA
one poly(dT)
two poly(dT)
two poly(dT) + Ribo-
[FU]
[s]
E
rC
RT: 0.55
Relative Abundance
Time (min)
y = 2E+06x + 2E+06
R² = 0.9981
pmol
m³C
RT: 0.88
y = 5864.2x - 13961
R² = 0.9993
m⁵C
RT: 1.06
y = 2825.2x + 5770.6
R² = 0.9971
hm⁵C
RT: 0.58
y = 2191.8x + 2596.2
R² = 0.9977
NH₂
HO
OH
CH₃
H₃C
F
m⁵C/m³U in 28S rRNA
**
WT
Tet2 KO

Figure S1. Purification of 18S rRNA, 28S rRNA, tRNA, and polyadenylated RNA, and LC-MS/MS quantification of C, m^3C, m^5C, and hm^5C in RNA. (**A**) Gel image of 18*S* rRNA and 28*S* rRNA. (**B**) Gel image of small RNAs (size smaller than 200 nt). (**C**) RNA bioanalyzer results of the purification process of polyadenylated RNAs. (**D**) This gel-like image is the stimulated image of the transfer of the raw migration time data to mimic electrophoretic assays. (**E**) LC-MS/MS channels, peak areas, and standard curves of cytidine, m^3C, m^5C, and hm^5C. RT: retention time. (**F**) LC-MS/MS detection of m^5C and m^3U in 28*S* rRNA extracted from wild type and *Tet2* KO mESCs (raw values without quantification with external standards). p values were determined using two-tailed Student's t-test for unpaired samples. Error bars represent mean ± s.d., n = 3 (three biological replicates × two technical replicates) **** $p < 0.001$

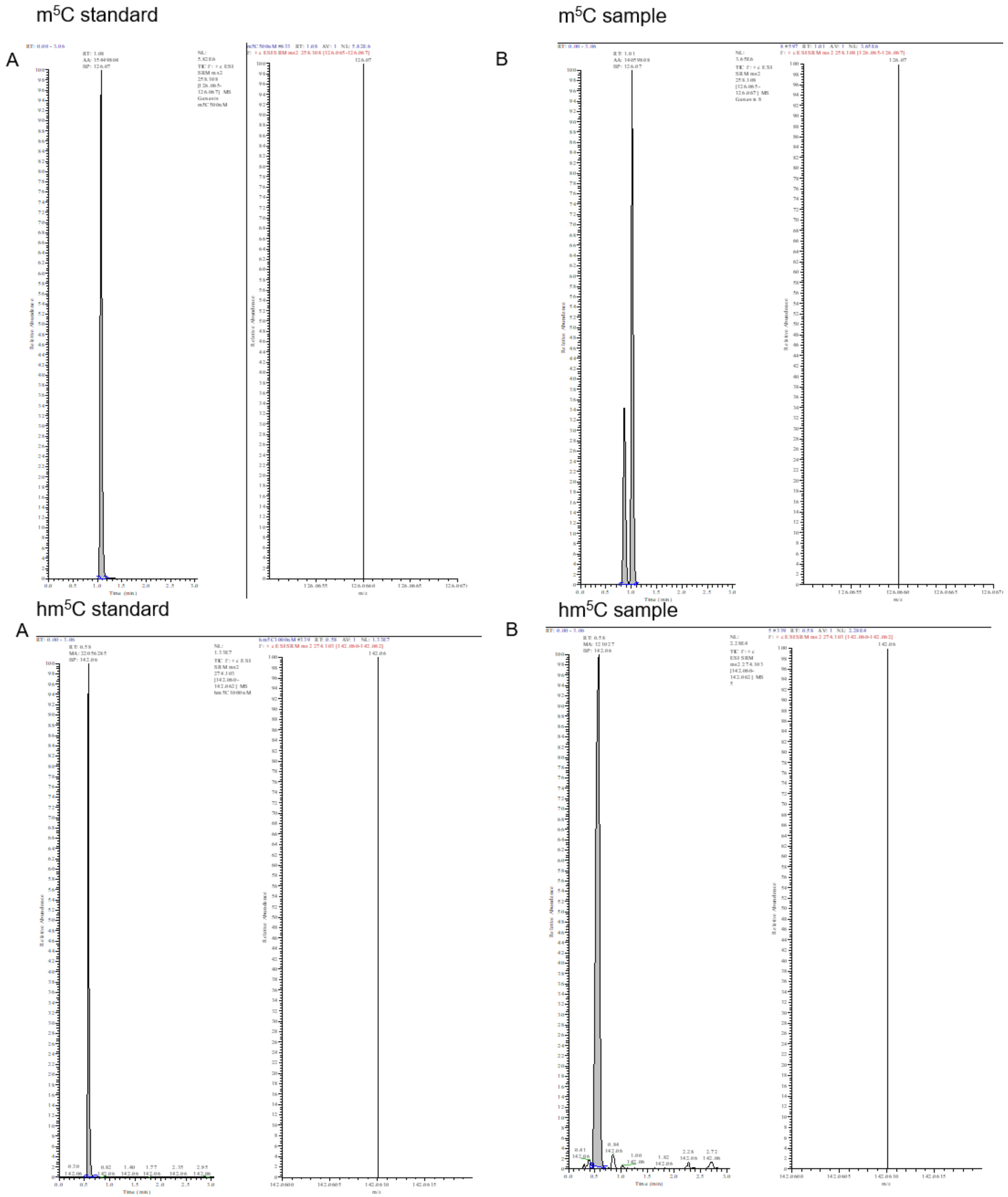

Figure S2. ESI-CID data for m^{5}C and hm^{5}C (paired with chromatogram peaks), measured using (A) synthetic standards and (B) tRNA isolated from TET2 + DOX HEK 293T cells.

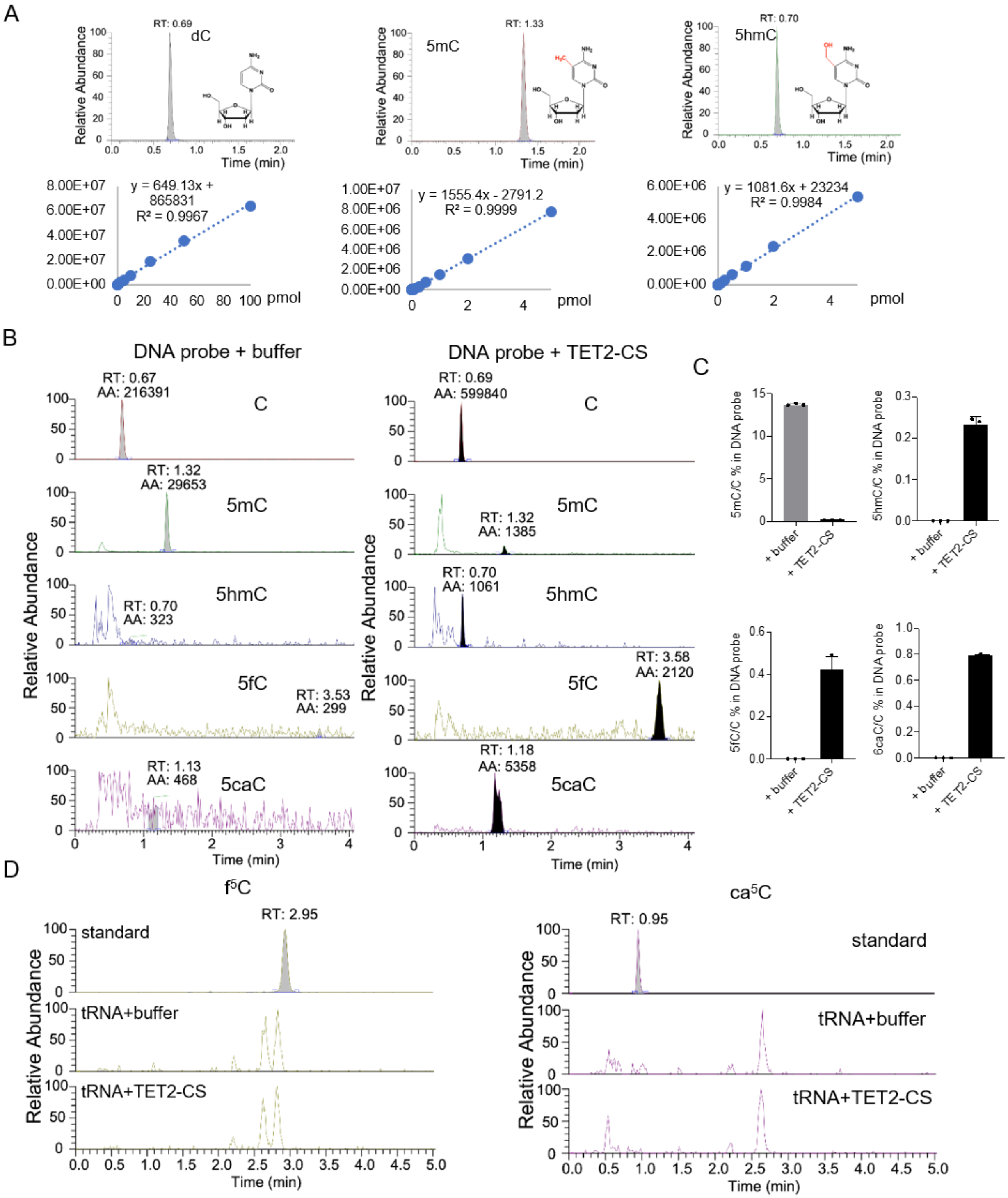

E

f5C		ca5C	
LOD (fmol)	2.3	LOD (fmol)	2.45
LOQ (fmol)	6.9	LOQ (fmol)	7.35

Figure S3. LC-MS/MS quantification of deoxycytidine, 5mC, 5hmC, 5fC, and 5caC in TET2-CS catalyzed *in vitro* oxidation reaction with a 5mC-containing DNA probe. (**A**) LC-MS/MS channels, peak areas, and standard curves of deoxycytidine, 5mC, and 5hmC. (**B**) LC-MS/MS peak areas of deoxycytidine, 5mC, 5hmC, 5fC, and 5caC in TET2-CS catalyzed *in vitro* oxidization reaction with a 5mC-containing DNA probe. LC-MS/MS quantification of (**C**) 5mC, 5hmC, 5fC, and 5caC in TET2-CS-mediated *in vitro* oxidation with a 5mC-containing DNA probe; and (**D**) f^5C and ca^5C in TET2-CS-mediated *in vitro* oxidation with purified tRNA. (**E**) Calculation of LOD and LOQ of 5fC and 5caC. Limit of detection (LOD) – lowest amount (in our case, of fmol) at which you can reliably say that you can detect an analyst (qualitatively). Limit of quantification (LOQ) – lowest amount (in our case, of fmol) at which you can reliably quantify an analyst. Concentrations below LOD = below detection limit. Concentrations between LOD and LOQ = below quantification limit, but definitely there Concentrations above LOQ = quantifiable.

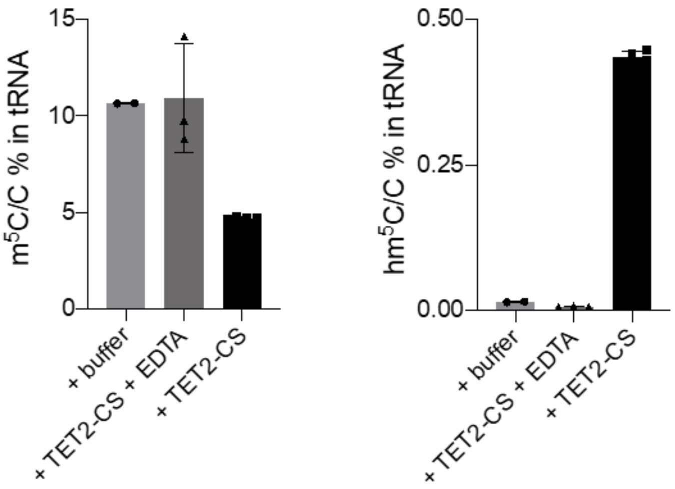

Figure S4. *In vitro* oxidation reaction of TET2-CS with purified tRNAs in the presence and absence of EDTA. *p* values were determined using two-tailed Student's t-test for unpaired samples. Error bars represent mean ± s.d., n = 3 (three biological replicates × two technical replicates).

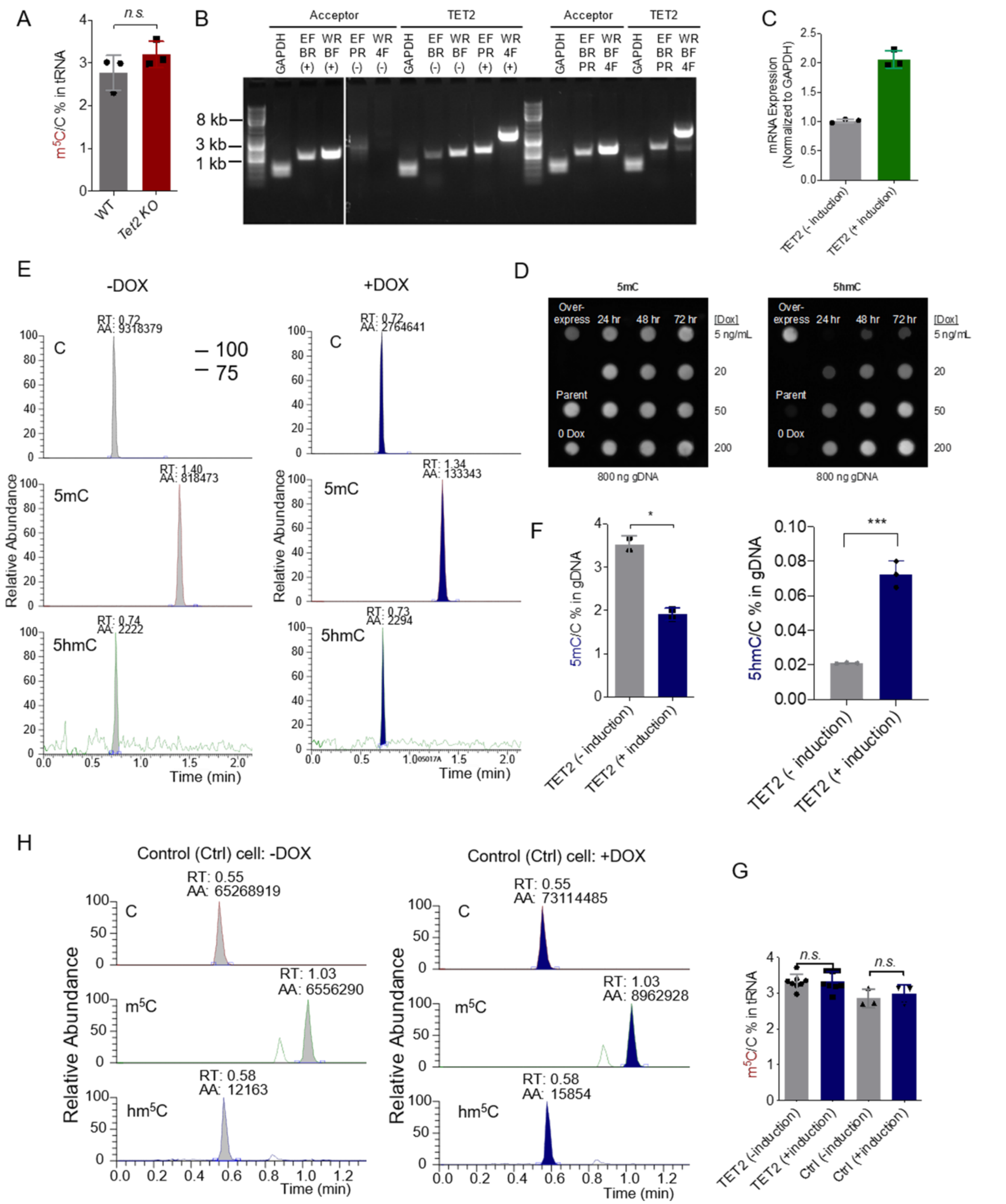
A
n.s.
m^5C/C % in tRNA
WT
Tet2 KO
B
Acceptor
TET2
Acceptor
TET2
GAPDH
EF BR (+)
WR BF (+)
EF PR (-)
WR 4F (-)
EF BR (-)
WR BF (-)
EF PR (+)
WR 4F (+)
EF BR PR
WR BF 4F
8 kb
3 kb
1 kb
C
mRNA Expression (Normalized to GAPDH)
TET2 (- induction)
TET2 (+ induction)
E
-DOX
+DOX
RT: 0.72
AA: 9318379
AA: 2764641
C
5mC
5hmC
RT: 1.40
AA: 818473
RT: 1.34
AA: 133343
RT: 0.74
AA: 2222
RT: 0.73
AA: 2294
100
75
Relative Abundance
Time (min)
D
5mC
5hmC
Over-express
24 hr
48 hr
72 hr
[Dox]
5 ng/mL
20
50
200
Parent
0 Dox
800 ng gDNA
F
5mC/C % in gDNA
5hmC/C % in gDNA
TET2 (- induction)
TET2 (+ induction)
*
***
H
Control (Ctrl) cell: -DOX
Control (Ctrl) cell: +DOX
RT: 0.55
AA: 65268919
AA: 73114485
C
m^5C
hm^5C
RT: 1.03
AA: 6556290
AA: 8962928
RT: 0.58
AA: 12163
AA: 15854
Relative Abundance
Time (min)
G
n.s.
m^5C/C % in tRNA
TET2 (-induction)
TET2 (+induction)
Ctrl (-induction)
Ctrl (+induction)

Figure S5. Construction and validation of TET2-CD-inducible expression cell lines. (**A**) LC-MS/MS quantification of m^5C in tRNA extracted from *Tet2* KO and wild type mESCs. (**B**) Gel showing amplification products from PCR on genomic DNA from acceptor HEK293T cells compared to cells containing the TET2 cassette. The TET2 stable cell line exhibits the expected shifts in PCR amplification products. (**C**) Quantification of the TET2 mRNA level before and after induction by qPCR. The expression level was normalized to GAPDH. (**D**) To test inducible expression of TET2-CD from stable cell lines, cells were split into 6-well plates. The next day, with the cells at 60-70% confluency, doxycycline was added to each well to a final concentration of 5-200 ng/mL. Cells were harvested at 24, 48, or 72 hours. Genomic DNA was purified from each batch of cells, and dot blotting was performed to detect 5mC and 5hmC. Regular HEK293T cells overexpressing TET2 were used as a positive control (top left corner), while parent acceptor cells (lacking the TET2-CD locus) and TET2-CD inducible cells without doxycycline induction were used as negative controls (bottom left corner). (**E**) LC-MS/MS tracks and (**F**) quantification 5mC and 5hmC in genomic DNA extracted from TET2 inducible expression of HEK293T cells before and after doxycycline induction. (**G**) LC-MS/MS quantification of m^5C in tRNAs extracted from TET2 inducible cells and control cells before and after doxycycline treatment. (**H**) LC-MS/MS peak areas of m^5C and hm^5C in tRNA of control HEK293T cells before and after doxycycline induction. RT: retention time. AA: atomic absorption. p values were determined using two-tailed Student's t-test for unpaired samples. Error bars represent mean ± s.d., n = 4 (four biological replicates × two technical replicates) *n.s.* means $p > 0.05$.

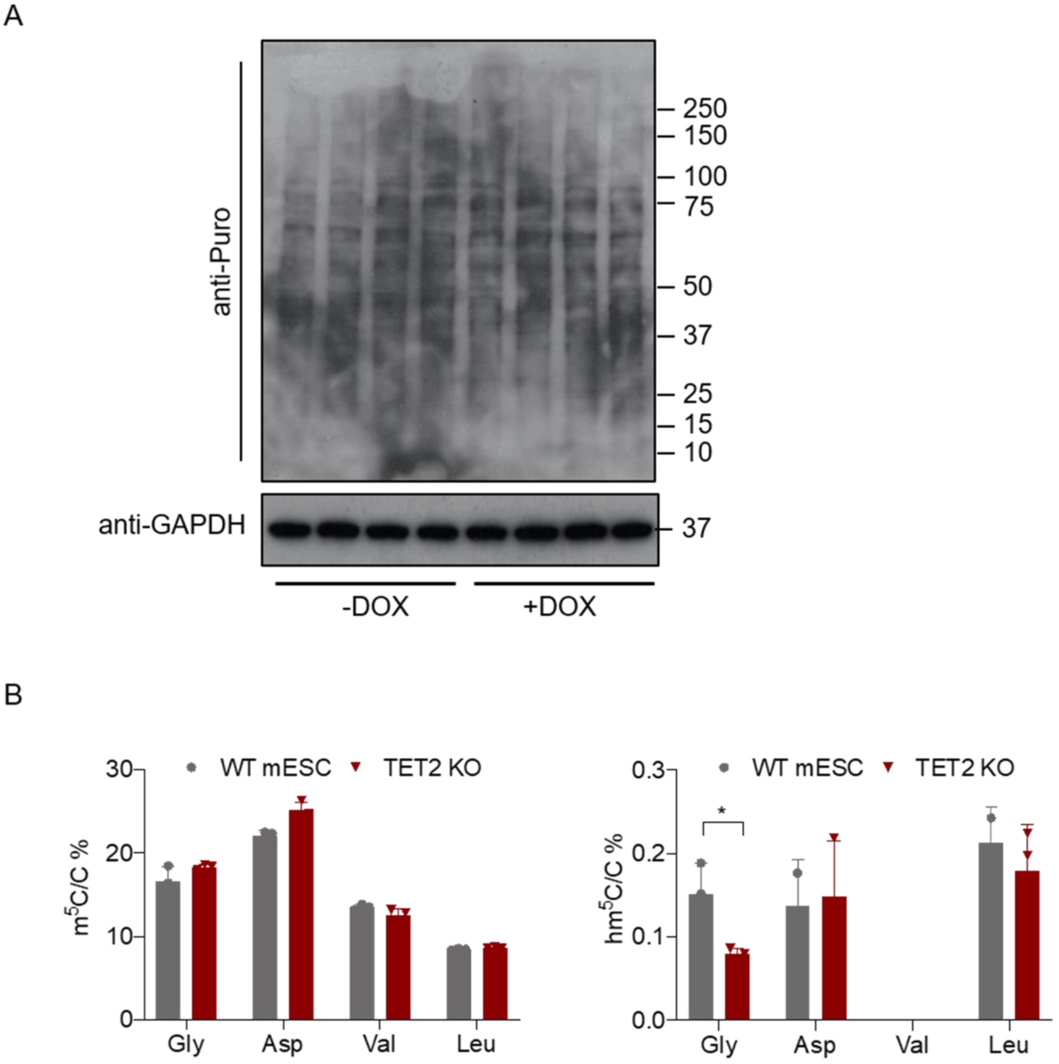

Figure S6. (**A**) Protein synthesis quantified using puromycin incorporation followed by Western blot analyses. First four lanes are from cell lysates of HEK 293T cells without DOX induction and the last four lanes are from cell lysates of HEK 293T cells after DOX-induced TET2 expression. The error bars showing the integrated signals from each Western blot sample in the image quantified by ImageJ. *p* values were determined using a two-tailed Student's t test for unpaired samples. Error bars represent mean ± SD, n = 4 (four biological replicates × two technical replicates). (**B**) LC-MS/MS quantification of m^5C in the individual tRNAs extracted from wild type and *Tet2* KO mESCs. *p* values were determined using two-tailed Student's t-test for unpaired samples. Error bars represent mean ± s.d., n = 2 (twp biological replicates × two technical replicates) * $p < 0.05$
